# Development and validation of a risk score in acute myocardial infarction-related cardiogenic shock

**DOI:** 10.1093/ehjacc/zuaf043

**Published:** 2025-03-25

**Authors:** Elma J Peters, Joakim B Kunkel, Margriet Bogerd, Sanne ten Berg, Marijke J C Timmermans, Ole K L Helgestad, Hanne B Ravn, Adriaan O Kraaijeveld, Luuk C Otterspoor, Krischan D Sjauw, Erik Lipšic, Annemarie E Engström, Alexander P J Vlaar, Christian Hassager, Jacob E Møller, José P S Henriques

**Affiliations:** Department of Cardiology, Heart Center, Amsterdam University Medical Center, Meibergdreef 9, Amsterdam 1105 AZ, The Netherlands; Department of Cardiology, Copenhagen University Hospital, Rigshospitalet, Copenhagen, Denmark; Department of Cardiology, Heart Center, Amsterdam University Medical Center, Meibergdreef 9, Amsterdam 1105 AZ, The Netherlands; Department of Cardiology, Heart Center, Amsterdam University Medical Center, Meibergdreef 9, Amsterdam 1105 AZ, The Netherlands; Netherlands Heart Registration, Utrecht, The Netherlands; Department of Cardiology, Odense University Hospital, Odense, Denmark; Department of Cardiothoracic Anesthesia, Odense University Hospital, Odense, Denmark; Department of Cardiology, Utrecht University Medical Center, Utrecht, The Netherlands; Department of Cardiology, Catharina Hospital, Eindhoven, The Netherlands; Department of Intensive Care, Catharina Hospital, Eindhoven, The Netherlands; Department of Cardiology, Medical Center Leeuwarden, Leeuwarden, The Netherlands; Department of Cardiology, St. Antonius Hospital, Nieuwegein, The Netherlands; Department of Cardiology, University Medical Center Groningen, Groningen, The Netherlands; Department of Intensive Care, Amsterdam University Medical Centers, Amsterdam, The Netherlands; Department of Intensive Care, Amsterdam University Medical Centers, Amsterdam, The Netherlands; Department of Cardiology, Copenhagen University Hospital, Rigshospitalet, Copenhagen, Denmark; Department of Cardiology, Copenhagen University Hospital, Rigshospitalet, Copenhagen, Denmark; Department of Cardiology, Odense University Hospital, Odense, Denmark; Department of Cardiology, Heart Center, Amsterdam University Medical Center, Meibergdreef 9, Amsterdam 1105 AZ, The Netherlands

**Keywords:** Cardiogenic shock, Mortality, Risk prediction, Prediction model, Acute myocardial infarction

## Abstract

**Aims:**

Mortality in patients with acute myocardial infarction-related cardiogenic shock (AMICS) is high, but a widely accepted tool for individual risk assessment is lacking. A reliable prediction model could assist in clinical decision-making, patient selection for clinical trials, and comparison of AMICS populations. Therefore, the aim of this study was to develop and externally validate a prediction model for 30-day mortality in AMICS patients.

**Methods and results:**

This retrospective cohort study included patients from 2017 to 2021 (development cohort) and 2010–2017 (validation cohort). Patients with AMICS undergoing percutaneous coronary intervention in The Netherlands were identified using the Netherlands Heart Registration. International validation was performed in the Danish Retroshock registry. The main outcome was 30-day mortality. Among 2261 patients, the median age was 67 years [interquartile range (IQR) 58–75], and 1649 (73%) were male. The mortality rate at 30 days was 39% (*n* = 886). Significant predictors for mortality were: initial lactate, glucose, renal function, haemoglobin, age, blood pressure, heart rate, intubation prior to PCI, intervention in the left main coronary artery, and successful revascularization. The AUC of the initial model was 0.81 (0.79–0.83). The external validation cohort included 1393 patients with 1050 (75%) male and a median age of 67 years (IQR 59–75). The 30-day mortality rate was 49% (*n* = 680). The model showed good performance on the external validation with an AUC of 0.73 (0.70–0.76).

**Conclusion:**

A prediction model was developed and externally validated using data from two large national registries. The model demonstrated good performance and is suitable for clinical decision-making and quality purposes in AMICS.

## Graphical Abstract

**Graphical Abstract:**
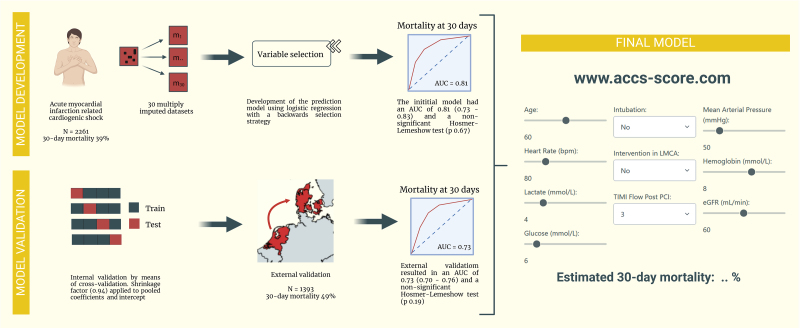
Created with Biorender.com.

## Introduction

Cardiogenic shock (CS) is a clinical syndrome characterized by hypotension and signs of organ hypoperfusion and is frequently caused by acute myocardial infarction (AMI).^1–3^ Even though the incidence of AMI-related cardiogenic shock (AMICS) only ranges from 3–13%, it is the leading cause of death in AMI worldwide with a 30-day mortality rate of around 40–50%.^3–5^

Acute myocardial infarction-related cardiogenic shock (AMICS) presents in many phenotypes and individualized assessment of each patient's risk could possibly assist in clinical decision-making for escalation in, e.g. medical therapy or mechanical circulatory support.^6^ Clinical trials in the acute setting of AMICS greatly suffer from difficulty in selecting patients as they present in a large variety of disease severity.^7^ Individualized selection could be enhanced by a widely accepted risk stratification tool. It would allow for identification of patients with a low risk of mortality but also of those with advanced shock and minimal chance of survival who would not benefit from more advanced therapy.

Since the 1990s, CS prediction models or severity scoring systems have been proposed for clinical practice.^8–18^ However, they were often based on mixed CS populations, included only a small number of patients, or were developed in a selected clinical trial population. Additionally, many of these models relied solely on clinical parameters, overlooking the importance of biochemical and angiographic factors.^19^ This limits the applicability of the existing scores. Hence, a multi-centre observational cohort study was conducted in AMICS patients using real-world data from a Dutch national registry. In this study, clinical, biochemical, angiographic, and therapeutic characteristics were assessed for their prognostic value for mortality in this severe clinical condition.^20^ The objective of the present study was to develop and externally validate a prediction model to estimate 30-day mortality in AMICS patients.

## Methods

### Study population and data collection

Patient data were retrieved from the Netherlands Heart Registration, a prospective registry in which data on all percutaneous coronary interventions (PCIs) in The Netherlands are collected.^21^ Patients with CS who underwent PCI were identified using this registry. Additional in-depth data were collected from AMICS patients who underwent PCI between January 2017 and September 2021 in 14 participating hospitals in The Netherlands (see Supplementary material online, *Table S1*). Details on the establishment and data collection process of this additional shock registry have been described in detail elsewhere.^20^ No ethical approval was required under the Medical Research Involving Human Subjects Act (WMO) as was confirmed by the Medical research Ethics Committees United (MEC-U). For the validation cohort, patients with AMICS treated by PCI in two centres between January 2010 and December 2017 were identified in the Danish Retroshock cohort.^22^

### Definitions

*Cardiogenic shock* was defined as: the presence of hypotension [systolic blood pressure (SBP) ≤ 90 mmHg for ≥30 min or support to maintain SBP ≥ 90 mmHg] and end-organ hypoperfusion (cold extremities and/or oliguria < 30 mL/h and/or heart rate ≥ 60 beats per minute). The endpoint *all-cause mortality at 30 days* was defined as whether or not a person was alive on day 30 after PCI. This was reliably obtained from the Personal Records Database (in Dutch: *Basisregistratie Personen*). All other definitions can be found in the data dictionary in Supplementary material online, *Table S3*. The definition for CS in the validation cohort was previously described by Helgestad *et al.*, and was based on ICD-10 codes in combination with treatment in the intensive care unit (ICU) and/or with vasoactive drugs and/or a mechanical assist device.^22^

### Statistical methods

#### General

Results are presented as numbers (*n*) and percentages (%), means and standard deviations (SD), or median and interquartile range (IQR) for variables with a skewed distribution. Comparisons were performed using Student's *t*-test, Mann–Whitney *U* test, or χ^2^ test, as appropriate. A total of 30 multiple imputed datasets was generated to account for missing data (see Missing data and sample size).

#### Model development

Multivariable logistic regression was performed on the imputed datasets with 30-day mortality as the dependent variable to select significant predictors. Three different models were developed at three different time-settings in patient care. The first model only included variables that were available at hospital admission. The second (and main) model included all variables that were available at the end of the PCI procedure. Lastly, the third model included all details from presentation, procedure and ICU or coronary care unit (CCU) admission. Left ventricular ejection fraction (LVEF) was also considered as a candidate predictor for the third model due to its deemed importance, despite its percentage missing^14^ (see Missing data and sample size). Duplicates or different variables expressing the same underlying clinical parameter were removed (e.g. creatinine and dialysis). Continuous variables were checked for linearity and included either as a continuous variable (when appropriate) or as a restricted cubic spline.^23^ Categorical variables were included as such, with merging of categories where appropriate (based on cell frequencies, clinical relevance, and predictive properties). Interaction terms or clinically relevant interactions were included. For the selection of variables, a backward stepwise selection strategy with a significance level value of 0.05 was used. A continued backwards selection was applied to come to a maximum of 10 predictors.

The collaborative consortium deemed the second model to be the most clinically relevant. This model included all variables up to and including the end of the PCI procedure. This model will be referred to as the main model in this paper. It will be presented in various formats, each designed to meet specific needs. Firstly, the regression formula will be provided, allowing users to estimate predicted mortalities for entire cohorts when used with statistical software. This formula could also be integrated into electronic patient records to automatically calculate individual patient risks. Secondly, a nomogram will be offered, giving a quick visual representation of the variables in the models, along with their ranges and relative importance. Lastly, a web-based calculator will be created to accurately compute individual risks in a clinical context.

#### Model validation

The developed models were internally validated through five-fold cross-validation with 10 iterations. We calculated the slope value from the cross-validation process to shrink the intercept and coefficients to minimize overfitting.^24^ The adapted models were then externally validated in Danish Retroshock data (see Supplementary material online, *Table S2*). The models’ performance was assessed by indices of discrimination and calibration. To assess model discrimination, the C-statistics with corresponding 95% confidence intervals (CIs) were calculated from the area under the receiver operating characteristic (ROC) curves (AUC). The model calibration was visually assessed by means of a calibration plot and tested with the Hosmer–Lemeshow goodness-of-fit test (where a non-significant test at the 0.05 level reflects a good model-fit).

#### Missing data and sample size

Regression analysis was performed after conducting a missing data analysis and applying multiple imputation. All variables with <40% missing data in the development cohort were included in the imputation. The outcome variable 30-day mortality was only included as a constraint variable, and missing fields were not filled. A total of 30 imputed datasets were generated with the mice package by means of predictive mean matching (mice, van Buuren *et al.*, v. 3.15.0, 2011). This was done separately for both the development and validation cohorts. The final results were subsequently obtained by pooling using Rubins’ Rules.^25^ A sample size calculation using the criteria proposed by Riley *et al*. was carried out to assess the number of variables that could be included in the model (pmsampsize, Ensor *et al.*, v. 1.1.2, 2022).^26^ Based on an expected C-statistic of 0.74 (derived from the IABP-SHOCK II score) and an outcome prevalence of 0.39 (as observed in our own data), the minimum required sample size was 2233 with 19.4 events per variable.^10^

All analyses were performed using R 4.2.1. (2022, Vienna, Austria) with the psfmi package (psfmi, Heymans, v. 1.1.0, 2022).

## Results

### Study population

A total of 2274 consecutive patients were identified that had CS and underwent PCI for AMI. Survival status at 30 days was unknown for 13 patients. Therefore, the final (Dutch) derivation cohort consisted of 2261 patients with an all-cause mortality at 30 days of 39% (*n* = 886) thereby meeting the required sample size. The Danish validation cohort consisted of 1393 patients with an all-cause mortality rate of 49% (*n* = 680). The characteristics of both populations are presented in *Table 1*. The cohorts were comparable in terms of age (median 67), male patient rate (around 74%), the prevalence of diabetes (around 20%), and a prior coronary event (27%). The levels of lactate, glucose, haemoglobin, and estimated glomerular filtration rate (eGFR) were also comparable at baseline. Some difference was observed in the rate of out-of-hospital cardiac arrest (OHCA), ST-segment elevated myocardial infarction (STEMI) vs. non-STEMI and intubation prior to PCI (41% vs. 58%, 86% vs. 72%, and 45% vs. 82% in development and validation data, respectively). The amount of missing data per variable are provided in Supplementary material online, *Figure S1*.

**Table 1:** Baseline characteristics

Development cohort						Validation cohort				
	Overall	Survivor	Deceased	*P*-value	Missing fraction	Overall	Survivor	Deceased	*P*-value	Missing fraction
*n*	2261	1375	886			1393	713	680		
Age (median [IQR])	67 [58, 75]	65 [57, 73]	71 [62, 78]	<0.001	0	67 [59, 75]	64 [55, 72]	71 [62, 79]	<0.001	0
Male sex (%)	612 (27.1)	367 (26.7)	245 (27.7)	0.65	0	1050 (75.4)	563 (79.0)	487 (71.6)	0.002	0
BMI (median [IQR])	26.1 [23.9, 29.1]	26.0 [23.7, 28.7]	26.2 [24.2, 29.6]	0.009	18.7	25.6 [23.5, 28.4]	25.8 [23.9, 28.7]	25.2 [23.1, 27.8]	0.024	28.6
Diabetes (%)	447 (20.8)	217 (16.4)	230 (27.8)	<0.001	4.8	242 (18.3)	95 (13.7)	147 (23.3)	<0.001	4.8
Prior coronary event (%)	549 (26.9)	313 (25.1)	236 (29.6)	0.028	9.6	366 (27.2)	189 (27.0)	177 (27.5)	0.878	3.4
Multivessel disease (%)	1356 (60.5)	766 (56.3)	590 (67.0)	<0.001	0.9	741 (56.0)	362 (53.0)	379 (59.2)	0.026	5
LVEF (%)										
Very severely impaired	50 (4.2)	20 (2.8)	30 (6.1)	<0.001	47.2	465 (35.3)	192 (27.5)	273 (44.0)	<0.001	5.4
Severely impaired	430 (36.0)	199 (28.3)	231 (47.1)			328 (24.9)	198 (28.4)	130 (20.9)		
Moderately impaired	157 (13.1)	101 (14.3)	56 (11.4)			284 (21.5)	154 (22.1)	130 (20.9)		
Mildly impaired	407 (34.1)	271 (38.5)	136 (27.8)			224 (17.0)	141 (20.2)	83 (13.4)		
Normal	150 (12.6)	113 (16.1)	37 (7.6)			17 (1.3)	12 (1.7)	5 (0.8)		
STEMI (%)	1930 (86.1)	1193 (87.8)	737 (83.6)	0.006	0.9	988 (72.1)	543 (77.5)	445 (66.4)	<0.001	1.6
Chest symptoms (%)										
<3 h	1145 (59.0)	733 (60.9)	412 (55.9)	<0.001	14.2	508 (45.2)	293 (50.2)	215 (39.9)	0.005	19.4
3–6 h	212 (10.9)	147 (12.2)	65 (8.8)			281 (25.0)	141 (24.1)	140 (26.0)		
6–12 h	160 (8.2)	97 (8.1)	63 (8.5)			127 (11.3)	52 (8.9)	75 (13.9)		
12–24 h	111 (5.7)	67 (5.6)	44 (6.0)			93 (8.3)	45 (7.7)	48 (8.9)		
>24 h	312 (16.1)	159 (13.2)	153 (20.8)			114 (10.2)	53 (9.1)	61 (11.3)		
OHCA (%)	927 (41.2)	495 (36.2)	432 (48.9)	<0.001	0.5	797 (57.5)	451 (63.6)	346 (51.2)	<0.001	0.6
IHCA (%)	284 (12.7)	126 (9.3)	158 (18.0)	<0.001	0.9	115 (8.3)	66 (9.3)	49 (7.2)	0.196	0
MAP (median [IQR])	87 [71, 108]	90 [73, 111]	83 [68, 103]	<0.001	13.2	80 [67, 96]	80 [67, 97]	78 [64, 95]	0.041	11.3
Heart rate (median [IQR])	82 [63, 101]	80 [60, 100]	89 [70, 108]	<0.001	14.1	86 [70, 104]	84 [70, 100]	89 [70, 105]	0.026	11.6
Intubation pre-PCI (%)	1013 (45.2)	494 (36.3)	519 (59.1)	<0.001	0.9	859 (82.0)	462 (83.2)	397 (80.5)	0.289	24.8
Vasoactive medication pre-PCI (%)										
None	1114 (50.9)	810 (60.8)	304 (35.5)	<0.001	3.2	740 (53.8)	372 (52.5)	368 (55.2)	0.013	1.3
Inotropes	34 (1.6)	22 (1.7)	12 (1.4)			246 (17.9)	144 (20.3)	102 (15.3)		
Vasopressor	826 (37.8)	411 (30.9)	415 (48.5)			177 (12.9)	98 (13.8)	79 (11.8)		
Both	214 (9.8)	89 (6.7)	125 (14.6)			212 (15.4)	94 (13.3)	118 (17.7)		
Lactate (median [IQR])	5.6 [2.7, 9.4]	4.3 [2.1, 7.2]	7.9 [4.0, 11.4]	<0.001	34.6	5.2 [3.0, 9.2]	4.1 [2.5, 7.2]	6.6 [3.7, 10.7]	<0.001	17.9
Haemoglobin (median [IQR])	8.4 [7.4, 9.2]	8.5 [7.6, 9.3]	8.2 [7.1, 9.1]	<0.001	5.5	8.5 [7.5, 9.2]	8.6 [7.8, 9.3]	8.2 [7.3, 9.1]	<0.001	22
eGFR (median [IQR])	61 [49, 75]	65 [53, 80]	54 [40, 67]	<0.001	9.5	58 [45, 72]	62 [50, 76]	54 [39, 68]	<0.001	20.5
Glucose (median [IQR])	12.2 [8.8, 17.1]	10.8 [8.3, 14.8]	14.8 [10.4, 20.0]	<0.001	11.5	12.5 [9.4, 17.6]	12.4 [9.3, 16.6]	12.6 [9.6, 18.8]	0.084	52.8
Intervention in LCA (%)	1389 (67.9)	802 (63.9)	587 (74.2)	<0.001	9.5	854 (74.5)	450 (74.8)	404 (74.3)	0.904	17.7
Intervention in RCA (%)	769 (37.6)	520 (41.4)	249 (31.5)	<0.001	9.5	366 (31.9)	196 (32.6)	170 (31.2)	0.681	17.7
TIMI pre-PCI (%)										
0	1277 (67.1)	780 (66.7)	497 (67.8)	0.823	15.9	729 (57.1)	364 (55.3)	365 (59.0)	0.225	8.3
1	182 (9.6)	110 (9.4)	72 (9.8)			107 (8.4)	50 (7.6)	57 (9.2)		
2	205 (10.8)	132 (11.3)	73 (10.0)			149 (11.7)	81 (12.3)	68 (11.0)		
3	238 (12.5)	147 (12.6)	91 (12.4)			292 (22.9)	163 (24.8)	129 (20.8)		
TIMI post-PCI (%)										
0	113 (5.8)	33 (2.7)	80 (11.1)	<0.001	14	48 (3.8)	8 (1.2)	40 (6.5)	<0.001	8.7
1	66 (3.4)	19 (1.6)	47 (6.5)			35 (2.8)	8 (1.2)	27 (4.4)		
2	188 (9.7)	108 (8.8)	80 (11.1)			117 (9.2)	43 (6.6)	74 (12.0)		
3	1578 (81.1)	1065 (86.9)	513 (71.2)			1072 (84.3)	596 (91.0)	476 (77.1)		
Mechanical circulatory support (%)	523 (23.4)	237 (17.4)	286 (32.5)	<0.001	1.1	371 (26.7)	197 (27.7)	174 (25.6)	0.423	0.1

Baseline characteristics of the two cohorts. Age in years; BMI, body mass index, in kg/m^2^; LVEF, left ventricular ejection fraction (very severely impaired = LVEF < 20%, severely impaired = LVEF 20–30%, moderately impaired = LVEF 30–40%, mildly impaired = LVEF 40–50%, normal = LVEF > 50%); STEMI, ST-segment elevation myocardial infarction; duration of chest symptoms, amount of time between start symptoms and hospital presentation; OHCA, out-of-hospital cardiac arrest; IHCA, in-hospital cardiac arrest; heart rate in beats per minute; MAP, mean arterial pressure, in mmHg; lactate in mmol/L; haemoglobin in mmol/L; eGFR in mL/min; glucose in mmol/L; LCA, left coronary artery; RCA, right coronary artery; TIMI, thrombolysis in myocardial infarction flow.

### Main model development and specification

The predictors resulting from the regression are shown in *Table 2*. The strongest correlations were found for: intubation before PCI, thrombolysis in myocardial infarction (TIMI)-flow post-PCI, age, lactate levels and intervention in the left main coronary artery. Furthermore, lower eGFR, haemoglobin, and mean arterial pressure on admission were associated with 30-day mortality. All included variables with corresponding odds ratios are shown in *Table 2*. All details of the additional models can be found in Appendices 2 + 3.

**Table 2:** Odds ratios main model

Term	Odds ratio	95% confidence interval		*P*-value
(Intercept)	0.237	0.047	1.194	0.080
Intubation prior to PCI	2.265	1.825	2.812	<0.001
Left main intervention	1.554	1.183	2.042	0.005
TIMI flow post-PCI (reference 3)				
2	1.731	1.244	2.408	0.001
0/1	4.404	3.099	6.258	<0.001
Age	1.023	1.007	1.040	0.006
Age' [50–67–82]	1.017	0.998	1.037	0.077
Heart rate	1.014	1.005	1.023	0.003
Heart rate' [45–82–120]	0.995	0.986	1.005	0.308
Lactate	1.212	1.113	1.320	<0.001
Lactate' [1.3–4.5–12.7]	0.833	0.724	0.958	0.010
Glucose	1.038	0.983	1.096	0.183
Glucose' [7–12–22]	1.021	0.946	1.102	0.591
eGFR	0.969	0.961	0.978	<0.001
eGFR' [34–61–91]	1.022	1.014	1.029	<0.001
MAP	0.988	0.979	0.997	0.010
MAP' [58–87–127]	1.005	0.993	1.016	0.435
Haemoglobin	0.857	0.746	0.985	0.030
Haemoglobin' [6.4–8.4–9.9]	1.097	0.942	1.277	0.235

All variables denoted with an apostrophe (’) represent the restricted cubic spline with three knots at 10th, 50th, and 90th percentiles. Knot locations are displayed between square brackets.

### Validation and performance

The initial model showed a C-statistic of 0.81 (95% CI 0.79–0.83) and demonstrated an adequate fit to the data, as indicated by a non-significant Hosmer–Lemeshow test (*P*-value 0.67).

**Figure 1:**
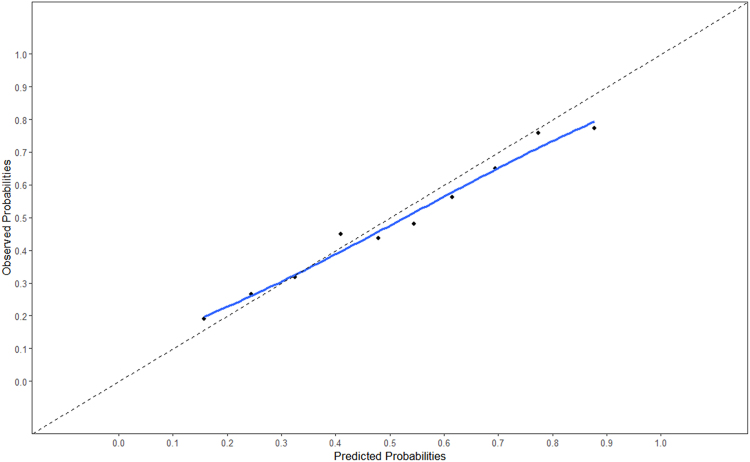
Calibration curve of the main model. Calibration curve of observed and predicted probabilities in external data of the main model.

Internal validation was performed by mean of cross-validation with three folds and five multiple imputation runs. The obtained shrinkage factor (0.89) was applied to the pooled coefficients and the intercept. The improved model was subsequently tested on the external dataset. The model's C-statistic in the external data was 0.73 (95% CI 0.70–0.76). The external calibration curve is shown in *Figure 1*.

### Usage in practice

**Figure 2:**
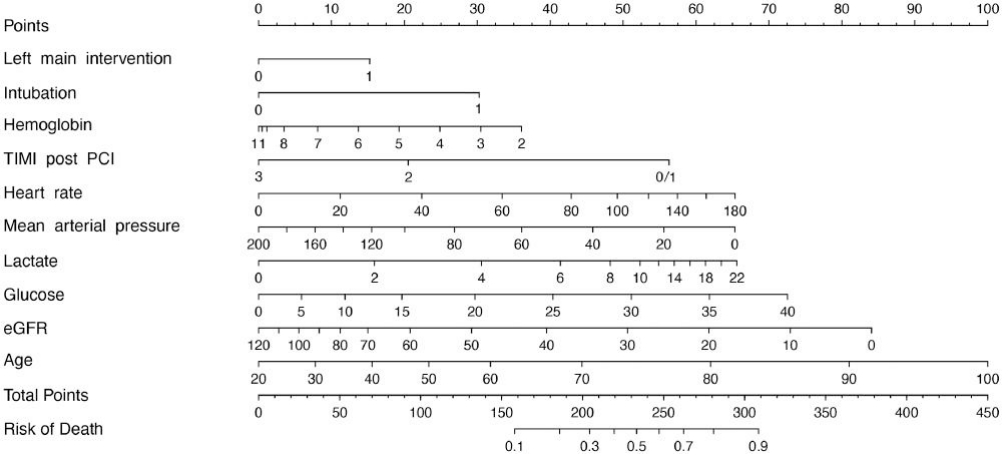
Nomogram to calculate risk predictions. For each variable, find the corresponding value on its axis. Then, draw a vertical line from that point upward to intersect with the ‘points scale’ at the top of the nomogram to find the number of points assigned based on the patient's value for that variable. Repeat this process for each variable in the nomogram. Sum all the assigned points to get the total score. Plot the total score on the ‘total points’ axis and draw a vertical line downward to intersect with the ‘risk of mortality’ scale at the bottom. This point represents the estimated risk of mortality for the patient.

We developed three practical applications for clinical usage:

1. The regression formula as shown in Appendix 3 and will be added to the digital supplementary files as an R object.
2. A nomogram (*Figure 2*) for a quick impression of an individual's prognosis but moreover to get an impression of the variables in the model including their ranges and weights.
3. A web-based calculator: www.accs-score.com. This is for individual risk assessment in clinical setting.

## Discussion

We developed and externally validated a prediction model for 30-day mortality in AMICS patients treated with PCI using data of two large national registries, yielding an AUC of 0.73 after external validation. The model included 10 predictors commonly collected in clinical practice, including patient-, laboratory-, and angiographic parameters.

The excellent performance of the model after internal validation, which was consistent with the initial performance, indicated its reliability and robustness. The external validation in data from a different country adds significant value to the findings. No other shock prediction tool was developed and validated in such large-sized cohorts and the currently developed risk score performs better than other contemporary scoring tools in comparable populations. Of the other scores developed specifically in CS following AMI, the IABP-shock II score is presumably the most widespread and validated score.^10^ It was developed in 480 patients from a randomized clinical trial and showed an AUC of 0.67 in a recent external validation study in 912 AMICS patients.^27^ A risk score developed by Klein *et al*. using registry data from AMI-related CS patients only included six variables and showed a C-statistic of 0.76. However, the development cohort of this score consisted of only 483 patients and external validation was omitted.^12^ An overview of other risk prediction tools including their parameters and discriminative abilities is provided in Supplementary material online, *Table S4*.

Both the Santiago Shock Score and the CardShock risk Score managed to achieve better discriminative performance, with C-statistics of 0.85 in their respective development cohorts.^14,17^ However, these models were developed in relatively small cohorts (135 and 219 patients, respectively) and included more heterogeneous populations. Given that different shock aetiologies have different prognoses, this increased heterogeneity is likely to lead to better discrimination.^28^ Lastly, the Society for Cardiovascular Angiography and Interventions (SCAI) shock stage classification has greatly helped the identification of various subgroups of shock severity since its development in 2019.^29^ It has been validated and is increasingly being used but is primarily a classification system rather than a prognostic tool for individualized risk assessment due to its broad categorization, limited set of clinical variables to define each stage, and absence of quantitative risk estimates.^30^ It may serve as a first clinical assessment whereas the currently developed tool may more exactly predict patients’ risk for death.

Many variables in our model are established risk factors also found in other scoring systems. However, our study uniquely assesses multiple predictors in a clearly defined, homogeneous group. All measured laboratory values (haemoglobin, lactate, glucose, eGFR) significantly predicted 30-day mortality. Lactate and eGFR, recognized markers of decreased tissue perfusion, are also used in other risk scores.^10,11,14,31^ Similarly, elevated glucose, or stress hyperglycaemia, is known to be associated with mortality in AMICS.^32^ Although haemoglobin level is not part of other CS risk scores, it is correlated with mortality in various conditions, emphasizing its importance in compromised haemodynamics.^33^

Out-of-hospital cardiac arrest was not independently associated with mortality in our large cohort, nor was it an effect-modifier in the relation between lactate and the outcome or vasoactive medication and the outcome. Literature on this topic is not conclusive but a trend towards higher mortality after OHCA in CS patients is described in most papers addressing this issue.^22,34^ We hypothesize that intubation before PCI and higher lactate and glucose levels may serve as surrogate markers for both IHCA and OHCA. Interestingly, parameters indicating comorbidities like diabetes, multivessel disease, and prior coronary events were largely insignificant predictors, thereby contrasting recent meta-analysis findings suggesting an increased mortality risk for diabetic patients with AMICS.^35^ These findings suggest acute disease markers may outweigh premorbid conditions in determining patient outcomes.

The current findings emphasize the role of several available markers in the risk assessment of CS. Decisions for escalation of therapy are often based on a crude clinical assessment including mainly patients’ age, whereas we have demonstrated that age is just one of the many factors with prognostic features. More specifically, markers of acute setting play at least an equal role and should be weighed in clinical decision-making accordingly.

### Strengths and limitations

Most importantly, this model was developed with high methodological accuracy. That is: (i) all candidate predictors could be included in the regression analysis as our cohort included a large number of patients with high outcome incidence; (ii) univariable selection was bypassed, and sample size criteria were easily met^36^; (iii) missing data were properly handled by multiple imputation; (iv) U-shaped relationships between mortality and markers were captured using restricted cubic splines^32,37^; and (v) external validation was performed on a real-world foreign cohort.

The model's practical applicability is enhanced by the use of objective, clearly defined variables available for almost every patient. This was crucial as we were unable to externally validate any of the existing scores in our current data; despite having over 100 variables present for every patient, all available scores contained unavailable parameters. Moreover, these variables can retrospectively be retrieved from records. By excluding subjective variables (e.g. confusion) and rarely measured biomarkers, this tool is particularly suited for quality of care and population comparisons.

Two real-world cohorts were used, with data manually collected to high standards. Only parameters available directly at the end of the PCI procedure were included in the main analysis, enabling early prediction.^38^ Finally, the patient cohort is clearly defined and homogeneous while covering several stages of shock, making it easy to determine which patients to apply the model to in practice.

The current study has some limitations as well. The model provides exact estimates when incorporated into electronic patient files or for users of the calculator, but it may be less suitable for mental calculations. Missing data, though handled by multiple imputation, remain a concern. No echocardiographic parameters were included in the main model despite the affirmed association with mortality in CS.^14,39^ Finally, inclusion periods of both cohorts differed ∼6 years, but we believe that this has minimal influence on the outcomes given the limited progress in terms of treatment or survival over the past years.

## Conclusion

We developed and externally validated a precise prediction model for 30-day mortality in patients with AMICS undergoing PCI. The model underscores the importance of several available markers in risk prediction and emphasizes that many factors should be taken into account when it comes to clinical decision-making. Besides use in clinical practice, the developed model will facilitate comparisons across different populations and assist in the selection of patients for clinical trials.

## Supplementary Material

zuaf043_Supplementary_Data

## Data Availability

The data presented in this study were obtained from national registries and are not openly available. Data may be provided upon request.

## Appendix 1

### Statistical methods

#### General

Results are presented as numbers (*n*) and percentages (%), means and standard deviations (SD), or median and interquartile range (IQR) for variables with a skewed distribution. Comparisons were performed using Student's *t*-test, Mann–Whitney *U* test or χ^2^ test, as appropriate. A total of 30 multiple imputed datasets was generated to account for missing data (see Missing data and sample size).

#### Model development

Multivariable logistic regression was performed on the imputed datasets with 30-day mortality as the dependent variable to select significant predictors. Three different models were developed at three different time-settings in patient care. The first model only included variables that were available at hospital admission. The second (and main) model included all variables that were available at the end of the PCI procedure. Lastly, the third model included all details from presentation, procedure and ICU or coronary care unit (CCU) admission. Left ventricular ejection fraction (LVEF) was also considered as a candidate predictor for the third model due to its deemed importance, despite its percentage missing^14^ (see Missing data and sample size). Duplicates or different variables expressing the same underlying clinical parameter were removed (e.g. creatinine and dialysis). Continuous variables were checked for linearity and included either as a continuous variable (when appropriate) or as a restricted cubic spline.^23^ Categorical variables were included as such, with merging of categories where appropriate (based on cell frequencies, clinical relevance and predictive properties). Interaction terms or clinically relevant interactions were included. For the selection of variables, a backward stepwise selection strategy with a significance level value of 0.05 was used. A continued backwards selection was applied to come to a maximum of 10 predictors.

The collaborative consortium deemed the second model to be the most clinically relevant. This model included all variables up to and including the end of the PCI procedure. This model will be referred to as the main model in this paper. It will be presented in various formats, each designed to meet specific needs. Firstly, the regression formula will be provided, allowing users to estimate predicted mortalities for entire cohorts when used with statistical software. This formula could also be integrated into electronic patient records to automatically calculate individual patient risks. Secondly, a nomogram will be offered, giving a quick visual representation of the variables in the models, along with their ranges and relative importance. Lastly, a web-based calculator will be created to accurately compute individual risks in a clinical context.

#### Model validation

The developed models were internally validated through five-fold cross-validation with 10 iterations. We calculated the slope value from the cross-validation process to shrink the intercept and coefficients to minimize overfitting.^24^ The adapted models were then externally validated in Danish Retroshock data (see Supplementary material online, *Table S2*). The models' performance was assessed by indices of discrimination and calibration. To assess model discrimination, the C-statistics with corresponding 95% confidence intervals (CIs) were calculated from the area under the receiver operating characteristic (ROC) curves (AUC). The model calibration was visually assessed by means of a calibration plot and tested with the Hosmer–Lemeshow goodness-of-fit test (where a non-significant test at the 0.05 level reflects a good model-fit).

#### Missing data and sample size

Regression analysis was performed after conducting a missing data analysis and applying multiple imputation. All variables with <40% missing data were included in the imputation. The outcome variable 30-day mortality was only included as a constraint variable, and missing fields were not filled. A total of 30 imputed datasets were generated with the mice package by means of predictive mean matching (mice, van Buuren *et al.*, v. 3.15.0, 2011). The final results were subsequently obtained by pooling using Rubins' Rules.^25^ A sample size calculation using the criteria proposed by Riley *et al*. was carried out to assess the number of variables that could be included in the model (pmsampsize, Ensor *et al.*, v. 1.1.2, 2022).^26^ Based on an expected C-statistic of 0.74 (derived from the IABP-SHOCK II score) and an outcome prevalence of 0.39 (as observed in our own data), the minimum required sample size was 2233 with 19.4 events per variable.^10^

All analyses were performed using R 4.2.1. (2022, Vienna, Austria) with the psfmi package (psfmi, Heymans, v. 1.1.0, 2022).

## Appendix 2

### Overview different models

**Table:**

	Model 1	Model 2	Model 3
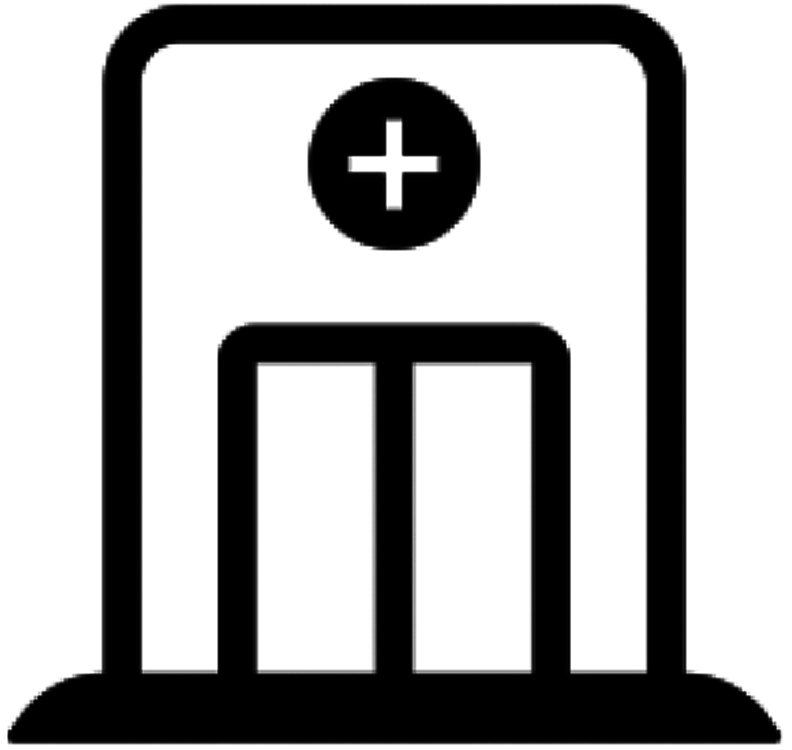
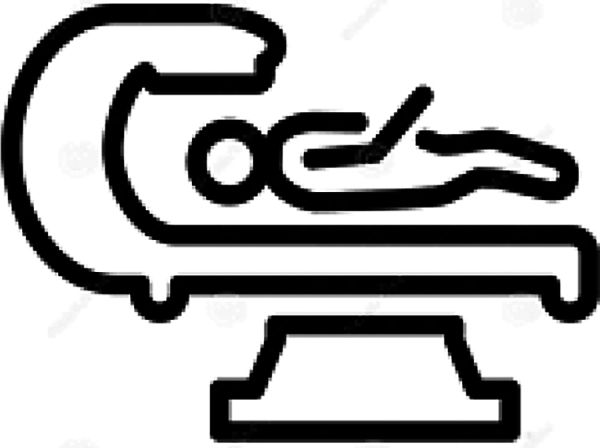
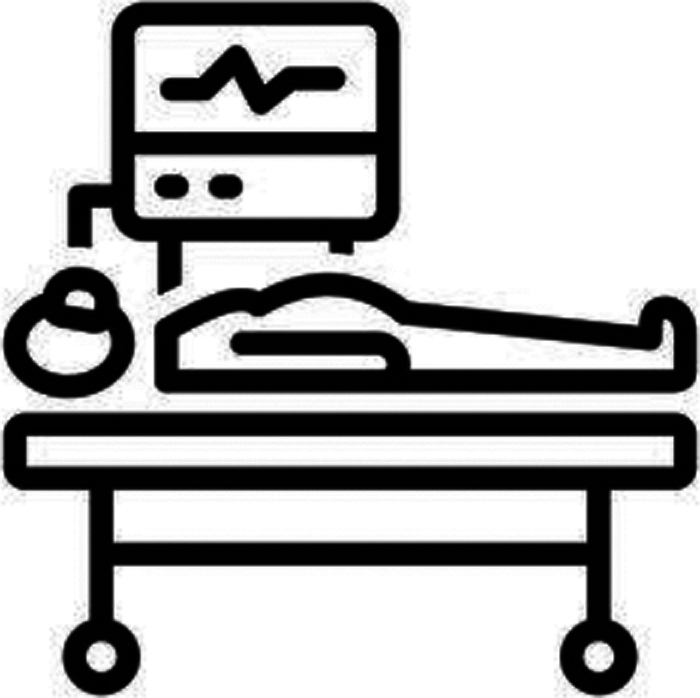
Intended use:	Upon hospital arrival Emergency Room	End of cath lab procedure Interventionalist	End of ICU/CCU admission Intensive care doctor
Variables considered for analysis	Age Sex BMI Diabetes Prior CE Chest symptom duration STEMI/NSTEMI Resuscitated Intubation Heart rate MAP Glucose eGFR Haemoglobin Lactate Interactions: Lactate: resuscitated Haemoglobin: sex	Age Sex BMI Diabetes Prior CE Chest symptom duration STEMI/NSTEMI Resuscitated Intubation Heart rate MAP Glucose eGFR Haemoglobin Lactate Inotropic pre-type TIMI flow pre-PCI TIMI flow post-PCI Multivessel disease Treated vessel Interactions: Lactate: resuscitated Haemoglobin: sex MAP: inotrope type Resuscitated: inotrope type	Age Sex BMI Diabetes Prior CE Chest symptom duration STEMI/NSTEMI Resuscitated Intubation Heart rate MAP Glucose eGFR Haemoglobin Lactate TIMI flow pre-PCI TIMI flow post-PCI Multivessel disease Treated vessel Number of inotropes during admission MCS during admission LVEF Interactions: Lactate: resuscitated Haemoglobin: sex MAP: inotrope type OHCA: inotrope type
Variables included	Age BMI STEMI/NSTEMI Chest symptom duration Heart rate Lactate Glucose eGFR Intubation MAP	Age Heart rate Lactate Glucose eGFR Intubation MAP TIMI post Haemoglobin Left main intervention	Age Lactate Glucose eGFR Intubation TIMI post MAP Inotropes MCS LVEF
Primary performance	AUC 0.80 (0.78–0.82) *R*^2^ 0.35 Hosmer–Lemeshow *P*-value 0.44	AUC 0.81 (0.80–0.84) *R*^2^ 0.38 Hosmer–Lemeshow *P*-value 0.57	AUC 0.84 (0.82–0.85) *R*^2^ 0.42 Hosmer–Lemeshow *P*-value 0.26
Shrinkage factor	0.94	0.89	0.89
After cross-validation	AUC 0.77 (95% CI 0.75–0.79) *R*^2^ 0.28	AUC 0.78 (95% CI 0.75–0.81) *R*^2^ 0.30	AUC 0.80 (95% CI 0.78–0.82) *R*^2^ 0.39
Performance external	AUC 0.72 (0.69–0.75) *R*^2^ 0.17	AUC 0.73 (0.70–0.76) *R*^2^ 0.20	AUC 0.73 (0.71–0.76) *R*^2^ 0.20

## Appendix 3

### 3A model 1

#### Odds ratios model 1

**Table:**

Term	OR	lower.EXP	upper.EXP	*P*-value
(Intercept)	0.370	0.056	2.438	0.301
STEMI aetiology	0.967	0.940	0.996	0.026
Intubation prior to PCI	2.053	1.646	2.561	<0.001
Chest symptoms (reference < 3 h)				
3–24 h	1.042	0.812	1.337	0.745
>24 h	1.606	1.193	2.162	0.002
Age	1.026	1.009	1.044	0.003
Age' [50–67–82]	1.022	1.002	1.043	0.028
Heart rate	1.015	1.005	1.024	0.002
Heart rate' [45–82–120]	0.995	0.985	1.004	0.279
Lactate	1.264	1.157	1.382	<0.001
Lactate' [1.3–4.5–12.7]	0.797	0.689	0.922	0.002
Haemoglobin	0.874	0.757	1.010	0.068
Haemoglobin' [6.4–8.4–9.9]	1.115	0.951	1.306	0.180
Glucose	1.040	0.983	1.101	0.170
Glucose' [7–12–22]	1.024	0.947	1.108	0.554
eGFR	0.971	0.962	0.981	<0.001
eGFR' [34–61–91]	1.021	1.013	1.029	<0.001
MAP	0.986	0.977	0.996	0.004
MAP' [58–87–127]	1.007	0.995	1.019	0.277

All variables denoted with an apostrophe (’) represent the restricted cubic spline with three knots at 10th, 50th, and 90th percentiles. Knot locations are displayed between square brackets.

#### Regression formula model 1

**Table:**

LnOdds(30-day mortality) = - 0.993 - 0.033 * STEMI + 0.719 * Intubation prior to PCI + 0.041 * Duration of symptoms (3-24 hours) + 0.474 * Duration of symptoms (> 24 hours) + 0.026 * Age + 0.022 * Age’[50-67-82] + 0.015 * Heart rate - 0.005 * Heart rate’[45-82-120] + 0.234 * Lactate - 0.227 * Lactate’ [1.3-4.5-12.7] - 0.134 * Hemoglobin + 0.109 * Hemoglobin’ [6.4–8.4–9.9] + 0.040 * Glucose + 0.024 * Glucose’[7-12-22] - 0.029 * eGFR + 0.020 * eGFR’ [34-61-91] - 0.014 * MAP + 0.007 * MAP’[59-87-127]

Reference for duration of symptoms is <3 h. All variables denoted with an apostrophe (’) represent the restricted cubic spline with three knots at 10th, 50th, and 90th percentiles. Knot locations are displayed between square brackets.

### 3B model 2

#### Odds ratios model 2

**Table:**

Term	OR	lower.EXP	upper.EXP	*P*-value
(Intercept)	0.237	0.047	1.194	0.080
Intubation prior to PCI	2.265	1.825	2.812	<0.001
Left main intervention	1.554	1.183	2.042	0.005
TIMI flow post-PCI (reference 3)				
2	1.731	1.244	2.408	0.001
0/1	4.404	3.099	6.258	<0.001
Age	1.023	1.007	1.040	0.006
Age' [50–67–82]	1.017	0.998	1.037	0.077
Heart rate	1.014	1.005	1.023	0.003
Heart rate' [45–82–120]	0.995	0.986	1.005	0.308
Lactate	1.212	1.113	1.320	<0.001
Lactate' [1.3–4.5–12.7]	0.833	0.724	0.958	0.010
Glucose	1.038	0.983	1.096	0.183
Glucose' [7–12–22]	1.021	0.946	1.102	0.591
eGFR	0.969	0.961	0.978	<0.001
eGFR' [34–61–91]	1.022	1.014	1.029	<0.001
MAP	0.988	0.979	0.997	0.010
MAP' [58–87–127]	1.005	0.993	1.016	0.435
Haemoglobin	0.857	0.746	0.985	0.030
Haemoglobin' [6.4–8.4–9.9]	1.097	0.942	1.277	0.235

All variables denoted with an apostrophe (’) represent the restricted cubic spline with three knots at 10th, 50th, and 90th percentiles. Knot locations are displayed between square brackets.

#### Regression formula model 2

**Table:**

LnOdds(30 day mortality) = - 1.438 + 0.818 * Intubation prior to PCI + 0.441 * Left main intervention + 0.549 * TIMI (2) + 1.482 * TIMI (0/1) + 0.023 * Age + 0.017 * Age' [50-67-82] + 0.014 * Heart rate -0.005 * Heart rate' [45-82-120] + 0.192 * Lactate - 0.182 * Lactate' [1.3-4.5-12.7] + 0.037 * Glucose + 0.021 * Glucose' [7-12-22] - 0.031 * eGFR 0.022 * eGFR' [34-61-91] - 0.012 * MAP + 0.005 * MAP'[58-87-127] - 0.154 * Hemoglobin + 0.092 * Hemoglobin'[6.4-8.4-9.9]

Reference value for TIMI post-PCI is 3. All variables denoted with an apostrophe (’) represent the restricted cubic spline with three knots at 10th, 50th, and 90th percentiles. Knot locations are displayed between square brackets.

### 3C model 3

#### Odds ratios model 3

**Table:**

Term	OR	lower.EXP	upper.EXP	*P*-value
(Intercept)	0.214	0.051	0.893	0.034
Intubation prior to PCI	2.044	1.625	2.570	<0.001
Mechanical circulatory support	1.605	1.272	2.025	<0.001
Left ventricular ejection fraction	0.975	0.964	0.986	<0.001
TIMI flow post-PCI (reference 3)				
2	1.624	1.162	2.270	0.005
0/1	4.734	3.323	6.744	<0.001
No. of inotropes (reference 0)				
1	1.609	1.115	2.322	0.011
≥2	2.785	1.952	3.973	<0.001
Age	1.026	1.008	1.043	0.003
Age' [50–67–82]	1.024	1.004	1.044	0.020
Lactate	1.165	1.070	1.268	<0.001
Lactate' [1.3–4.5–12.7]	0.859	0.746	0.988	0.034
Glucose	1.011	0.957	1.069	0.686
Glucose' [7–12–22]	1.050	0.972	1.135	0.218
eGFR	0.971	0.963	0.980	<0.001
eGFR' [34–61–91]	1.020	1.013	1.028	<0.001
MAP	0.991	0.982	1.000	0.048
MAP' [58–87–127]	1.004	0.992	1.015	0.545

All variables denoted with an apostrophe (’) represent the restricted cubic spline with three knots at 10th, 50th, and 90th percentiles. Knot locations are displayed between square brackets.

#### Regression formula model 3

**Table:**

LnOdds (30 day mortality) = -1.542 + 0.715 * Intubation prior to PCI + 0.473 * MCS - 0.025* LVEF + 0.485 * TIMI(2) + 1.555 * TIMI (0/1) + 0.476 * Inotropes during admission (1) + 1.024* Inotropes during admission (> 2) + 0.025 * Age + 0.023 * Age' [50-67-82] + 0.152 * Lactate - 0.153 * Lactate' [1.3-4.5-12.7] + 0.011 * Glucose + 0.049 * Glucose' [7-12-22] - 0.029 * eGFR + 0.020 * eGFR' [34-61-91] - 0.009 * MAP + 0.004 * MAP' [58-87-127]

Reference value for TIMI post-PCI is 3. Reference value for inotropes during admission is zero. All variables denoted with an apostrophe (’) represent the restricted cubic spline with three knots at 10th, 50th, and 90th percentiles. Knot locations are displayed between square brackets.
