# Supplementary material for: Development and validation of a risk score in acute myocardial infarction-related cardiogenic shock
Source: Eur Heart J Acute Cardiovasc Care. 2025 Mar 25;14(6):327–41. doi: 10.1093/ehjacc/zuaf043 (PMC12365599; doi:10.1093/ehjacc/zuaf043)
Supplement: zuaf043_Supplementary_Data [file zuaf043_Supplementary_Data.zip › Supplementary material.docx]

Supplementary material

CONTENTS

Supplementary table 1: Dutch consortium

Supplementary table 2: Danish consortium

Supplementary figure 1: Percentages missing data

Supplementary table 3: Data dictionary

Supplementary table 4: Overview of the prediction models covered

Supplementary table 1

Dutch consortium

| Hospital |  | Physician |
| --- | --- | --- |
| Amphia Hospital |  | Dr. M. Meuwissen |
| Amsterdam University Medical Center, AMC |  | Prof. dr. J.P. Henriques |
| Amsterdam University Medical Center, VUmc |  | Dr. C.J.W. Verouden |
| Catharina Hospital |  | Dr. L.C. Otterspoor  Dr. K. Teeuwen |
| Erasmus Medical Center |  | Drs. J.J.H. Bunge  Dr. E.A. Dubois |
| Haga Hospital |  | Dr. G.B. Bleeker |
| Isala hospital |  | Dr. I. Andrade Ferreira |
| Leiden University Medical Center |  | Drs. J. Montero-Cabezas |
| Medical Center Leeuwarden |  | Dr. K.D. Sjauw |
| Noordwest Clinics, Alkmaar |  | Drs. A. Dedic  Dr. J. van Ramshorst |
| Radboud University Medical Center |  | Prof. dr. R.J. van Geuns |
| Rijnstate Hospital |  | Dr. P.W. Danse |
| University Medical Center Groningen |  | Dr. E. Lipsic |
| University Medical Center Utrecht |  | Dr. A.O. Kraaijeveld |

Supplementary table 2

Danish consortium

| Hospital |  | Physician |
| --- | --- | --- |
| Copenhagen University Hospital – Rigshospitalet, Copenhagen, Denmark |  | Dr. Jakob Josiassen  Prof. dr. C. Hassager  Prof. dr. J. E. Møller  Prof. dr. L. Holmvang |
| Odense University Hospital, Odense, Denmark |  | Dr. O. K. L. Helgestad  Prof. dr. H. B. Ravn  Prof. dr. H. Schmidt  Prof. dr. L. O. Jensen |

| Supplementary figure 1  Percentages missing data | |
| --- | --- |
| 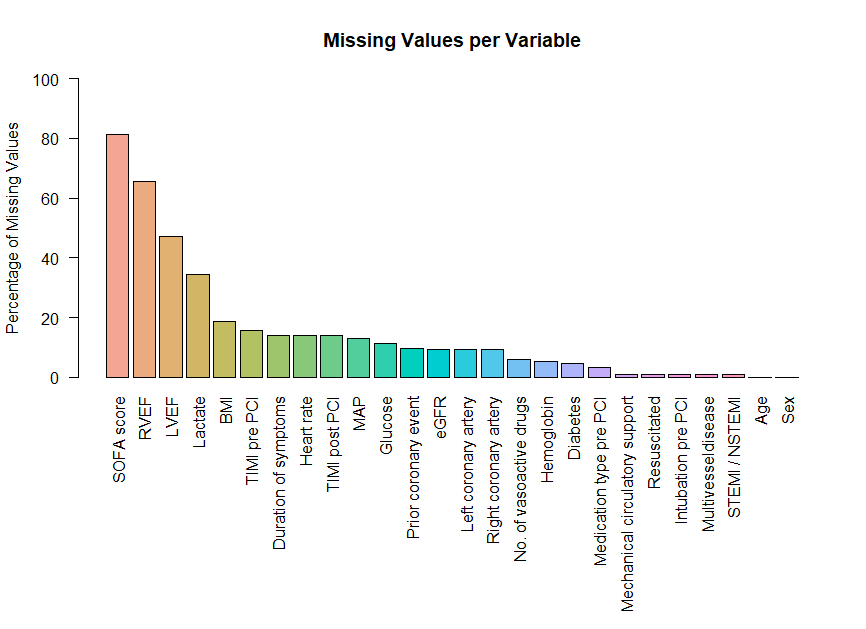 **A** | |
| 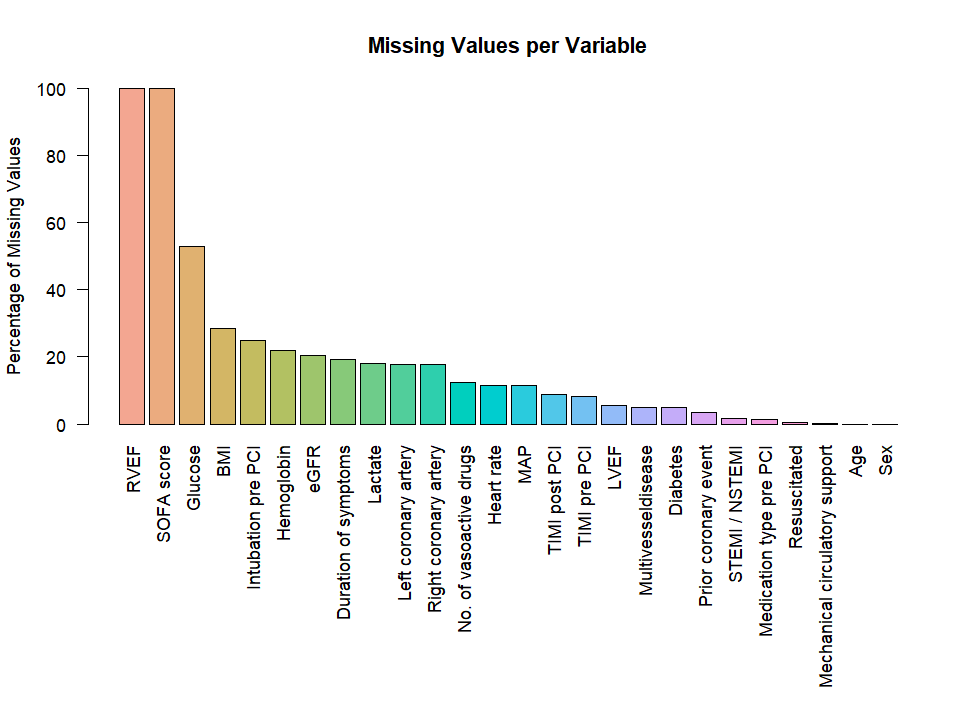**B** | |
|  | Percentages missing in Dutch (A) and Danish (B) data  **SOFA** = sequential organ failure assessment; **RVEF** = Right ventricular ejection fraction; **LVEF** = Left ventricular ejection fraction; **BMI** = Body mass index; **TIMI** = Thrombolysis in myocardial infarction flow; **PCI** = Percutaneous coronary intervention; **MAP** = Mean arterial pressure; **Coronary event** = prior PCI, myocardial infarction and/or coronary artery bypass grafting; **eGFR** = estimated glomerular filtration rate; **No. of vasoactive drugs** = Number of medications given from the following: adrenaline, noradrenaline, dobutamine, dopamine and enoximon/milrinon; **Medication type pre pci** = vasopressor, inotrope, combination or none given pre PCI; **(N)STEMI** = (Non) ST-segment elevated myocardial infarction. |

Supplementary table 3

Data dictionary

| Variable | Scale |
| --- | --- |
| Age  *Difference between date of birth and date of intervention* | Continuous |
| Sex  *A persons’ biological sex* | Male  Female |
| Height  *Most recently reported height, measured during index admission. When height is not measured during index admission, the most recently reported height, up to one year old, is used.* | Continuous |
| Weight  *Most recently reported weight, measured during index admission. When weight is not measured during index admission, the most recently reported weight, up to one year old, is used.* | Continuous |
| Body Mass Index  *Weight in kg / (height in meters)^2^* | Continuous |
| Diabetes mellitus  *Whether a person has diabetes or not, regardless of treatment status.* | No  Yes |
| Prior MI  *Patient had at least one documented prior myocardial infarction, excluding infarctions occurring during the same admission that are the reason for the current intervention.* | No  Yes |
| Prior PCI  *Patient underwent PCI prior to current intervention.* | No  Yes |
| Prior CABG  *Patient underwent coronary artery bypass grafting prior to current intervention.* | No  Yes |
| Shock etiology  *Status of the patient during the current intervention.*  *NSTEMI: presence of acute chest pain in the absence of ST-elevation (including stable angina)*  *STEMI: presence of acute chest pain and (>20mm) ST-elevation.* | NSTEMI  STEMI |
| Duration of chest symptoms  *Amount of time between start symptoms and hospital presentation.* | >24 hours  >12 hours, ≤24 hours  >6 hours, ≤12 hours  >3 hours, ≤6 hours  ≤3 hours |
| OHCA  *Patients who were defibrillated ( and received chest compressions) outside the hospital, prior to and related to the reason for the current intervention.* | No  Yes |
| IHCA  *Patient was defibrillated (and received chest compressions) in the hospital before entering the cath lab.* | No  Yes |
| Systolic blood pressure – mmHg  *Systolic blood pressure according to first in-hospital measurement pre-PCI. In case of absence of an in-hospital measurement, a measurement by the emergency medical team is used.* | Continuous |
| Diastolic blood pressure – mmHg  *Diastolic blood pressure according to first in-hospital measurement pre-PCI. In case of absence of an in-hospital measurement, a measurement by the emergency medical team is used.* | Continuous |
| Mean arterial pressure – mmHg  *The average arterial pressure throughout one cardiac cycle, calculated as*  *(2*diastolic blood pressure + systolic blood pressure) / 3* | Continuous |
| Heart rate – bpm  *Heart rate according to first in-hospital measurement pre-PCI. In case of absence of an in-hospital measurement, a measurement by the emergency medical team can be used.* | Continuous |
| Lactate on admission – mmol/L  *First measured blood lactate level on admission (±1 hour around PCI).* | Continuous |
| Hemoglobin on admission – mmol/L  *First measured hemoglobin level on admission (±1 hour around PCI).* | Continuous |
| Glucose on admission – mmol/L  *First measured glucose level on admission (±1 hour around PCI).* | Continuous |
| Creatinine on admission - µmol/L  *First measured creatinine level on admission (±1 hour around PCI).* | Continuous |
| Estimated glomerular filtration rate – ml/min/1.73m^2^  *Calculated using the MDRD formula using the first measured creatinine level on admission.* | Continuous |
| Intubation before PCI  *Patient is intubated prior to PCI (up to first pressure registration).* | No  Yes |
| Multivessel disease  *Presence of multivessel disease during the current intervention. For first interventions: stenosis of ≥70% in ≥2 native vessels with a diameter of at least 1.5 mm. In patients with a prior coronary intervention: ≥70% stenosis in ≥1 native coronary arteries that have not yet been treated and/or multivessel disease during previous intervention.* | No  Yes |
| TIMI flow grade pre-PCI  *TIMI-flow measured pre-PCI.* | 0  1  2  3 |
| TIMI flow grade post-PCI  *TIMI-flow measured post-PCI.* | 0  1  2  3 |
| PCI treated vessel  *Name of dilated coronary artery.*  *LM: left main*  *LAD: left coronary artery*  *RCX: circumflex artery*  *RCA: right coronary artery* | LM  LAD  RCX  RCA |
| Left ventricular ejection fraction (LVEF) - %  *Fraction of blood ejected from the left ventricle with each contraction, expressed as percentage, measured during shock. Most recent measure to be used, up to 2 hours before- and 24 hours after intervention. If more than one ejection fraction is available, lowest registered value should be registered.* | Continuous |
| Right ventricular ejection fraction (RVEF) – %  *Fraction of blood ejected from the right ventricle with each contraction, expressed as percentage, measured during shock. Most recent measure to be used, up to 2 hours before- and 24 hours after intervention. If more than one ejection fraction is available, lowest registered value should be registered.* | Continuous |
| Mechanical circulatory support  *Type of mechanical circulatory support that was initiated during index admission.* | None  IABP  Impella  ECMO  IABP + ECMO  Impella + ECMO  IABP + Impella  Other |
| Vasoactive agents pre PCI | None  Inotrope  Vasopressor  Both |
| Vasoactive medication during admission  Number of vasoactive agents that was administered somewhere during admission, from: noradrenaline, adrenaline, dobutamine, dopamine, milrinone / enoximone. | Continuous |

**Supplementary Table 4**

Overview of the prediction models covered

| **Name** | **Year** | **Sample size** | **Mortality** | **Population** | **Predictors** | **AUC** | **External validation** |
| --- | --- | --- | --- | --- | --- | --- | --- |
| **GUSTO prediction model (1)** | 1990-1993 | N = 2968  RCT | 30-Day mortality: 55% | AMI-CS | - Age - Height - Heart rate - Blood pressure - Time to thrombolytic treatment - Prior infarction - Prior angina - Infarction location - Killip class - Diabetes - Smoking status - Altered sensorium - Cold, clammy skin - Oliguria - Ventricular septal defect - Ventricular rupture - Arrythmia | AUC 0.82* | None |
| **SHOCK trial scoring system (2)** | 1993-1998 | N = 1217  RCT (294) + registry (923) | In-hospital mortality within 30 days: 57% | AMI-CS | - Age - Shock on admission - End-organ hypoperfusion - Anoxic brain damage - Systolic blood pressure - Prior CABG - Noninferior myocardial infarction - Creatinine ≥ 1.9 mg/dL | AUC 0.74 | None |
| **ACC-NCDR prediction model (3)** | 1998-2002 | N = 483  National registry | Overall in-hospital mortality: 59% | AMI-CS undergoing emergency PCI | - Age - History of renal insufficiency - Total occlusion in the LAD - No stent used - No GP IIb/IIIa used during PCI - Female sex | AUC 0.78 | None |
| **SAVE-score (4)** | 2003-2013 | N = 3846  Registry | Mortality at hospital discharge: 58% | Refractory CS (mixed etiology) treated with VA-ECMO | - Acute CS diagnosis group - Age - Weight - Acute pre-ECMO organ failures - Chronic renal failure - Duration of intubation prior to initiation of ECMO - Peak inspiratory pressure ≤20 cmH^2^O - Pre-ECMO cardiac arrest - Diastolic blood pressure before ECMO ≥ 40mmHg - Pulse pressure before ECMO ≤ 20mmHg - HCO_3_ before ECMO ≤15 mmol/L | 0.68 (0.64 – 0.71) | In Australian cohort (N = 161)  AUC = 0.90 (0.85 – 0.95) |
| **ENCOURAGE mortality risk score (5)** | 2008-2013 | N = 138  Prospective cohort | Mortality before ICU discharge: 53% | AMI-CS treated with VA-ECMO | - Age > 60 years - Female - BMI > 25 kg/m^2^ - Glasgow coma score < 6 - Creatinine >150 µmol/L - Serum lactate - Prothrombin activity < 50% | AUC 0.84 (0.77 – 0.91) | None |
| **IABP-SHOCK II risk score (6)** | 2009-2011 | N = 480  RCT | 30-Day mortality: 40% | AMI-CS | - Age > 73 years - History of stroke - Glucose > 10.6 mmol/L - Creatinine > 132.6 µmol/L - Arterial lactate >5mmol/K - TIMI flow grade < 3 after PCI | AUC 0.74 (0.69 – 0.78) | CardShock trial (N = 137)  AUC = 0.73 (0.64 – 0.81)  In CCCTN (N = 912 AMI-CS)  AUC = 0.67 |
| **Cardiogenic Shock Score (7)** | 2009-2019 | N = 1308  Registry | 30-day mortality: 57% | Mixed CS | - Age - Sex - Absence of AMI - Systolic blood pressure - Heart rate - pH - Lactate - Glucose - CPR | AUC 0.74 | Mixed CS patients (N = 934)  AUC 0.73 in AMI-CS |
| **CardShock risk Score (8)** | 2010-2012 | N = 219  Propspective observational trial | In-hospital mortality: 37% | Mixed CS | - Prior CABG - ACS etiology - Confusion - Previous MI - Blood lactate - LVEF - Age - Systolic blood pressure - eGFR | AUC 0.85 (0.79 – 0.90) | In IABP-SHOCK II trial (N = 384)  AUC 0.85 (0.80 -0.90) |
| **Santiago Shock Score (9)** | 2011-2020 | N = 135  Registry | In-hospital mortality: 41% | Mixed CS | - Age - Lactate - Mitral regurgitation grade - Hemoglobin - NT-propBNP - Etiology | AUC 0.85 (0.78 – 0.90) | Different cohort  (N = 113)  AUC 0.60 (0.50 – 0.71) |
| **Residual SYNTAX score (10)** | 2013-2017 | N = 706  RCT | 30-Day mortality: 47% | AMI-CS | - Remaining coronary stenoses following PCI |  | In CULPRIT-SHOCK trial (N = 587)  AUC 0.63 (0.59 – 0.68) |
| **CRASH score (11)** | 2016 | N=66  Prospective cohort | In hospital mortality: 30% | Mixed CS | - Cardiac power index - Inotropic score | AUC 0.85 | None |
| **Acute Coronary Syndrome Cardiogenic Shock Score (ACCS)** | 2017-2021 | N = 2261  Registry | 30-Day mortality: 39% | AMI-CS | - Age - Mean arterial pressure - Heart rate - Intubation before pre PCI - TIMI flow grade after PCI - Intervention in the LM - Lactate - Glucose - Hemoglobin - eGFR | AUC 0.81 (0.79 – 0.83) | In Danish Retroshock cohort (N = 1393)  AUC 0.73 (0.70–0.76) |
|  | RCT = Randomized controlled trial; AMI-CS = Acute myocardial infarction cardiogenic shock; CABG = Coronary artery bypass grafting; AUC = Area under the curve; ECMO = Extra corporeal membrane oxygenation; LAD = Left anterior descending coronary artery; TIMI = Thrombolysis in myocardial infarction flow grade; BMI = Body mass index; PCI = Percutaneous coronary intervention; LVEF = Left ventricular ejection fraction; eGFR = Estimated glomerular filtration rate; CPR = Cardiopulmonary resuscitation; LM = Left main coronary artery  * AUC of the regression model, not of the finalized scoring system | | | | | | |

1. Hasdai D, Holmes DR, Jr., Califf RM, Thompson TD, Hochman JS, Pfisterer M, Topol EJ. Cardiogenic shock complicating acute myocardial infarction: predictors of death. GUSTO Investigators. Global Utilization of Streptokinase and Tissue-Plasminogen Activator for Occluded Coronary Arteries. Am Heart J. 1999;138(1 Pt 1):21-31.

2. Sleeper LA, Reynolds HR, White HD, Webb JG, Dzavik V, Hochman JS. A severity scoring system for risk assessment of patients with cardiogenic shock: a report from the SHOCK Trial and Registry. Am Heart J. 2010;160(3):443-50.

3. Klein LW, Shaw RE, Krone RJ, Brindis RG, Anderson HV, Block PC, et al. Mortality after emergent percutaneous coronary intervention in cardiogenic shock secondary to acute myocardial infarction and usefulness of a mortality prediction model. Am J Cardiol. 2005;96(1):35-41.

4. Schmidt M, Burrell A, Roberts L, Bailey M, Sheldrake J, Rycus PT, et al. Predicting survival after ECMO for refractory cardiogenic shock: the survival after veno-arterial-ECMO (SAVE)-score. Eur Heart J. 2015;36(33):2246-56.

5. Muller G, Flecher E, Lebreton G, Luyt CE, Trouillet JL, Brechot N, et al. The ENCOURAGE mortality risk score and analysis of long-term outcomes after VA-ECMO for acute myocardial infarction with cardiogenic shock. Intensive Care Med. 2016;42(3):370-8.

6. Poss J, Koster J, Fuernau G, Eitel I, de Waha S, Ouarrak T, et al. Risk Stratification for Patients in Cardiogenic Shock After Acute Myocardial Infarction. J Am Coll Cardiol. 2017;69(15):1913-20.

7. Beer BN, Jentzer JC, Weimann J, Dabboura S, Yan I, Sundermeyer J, et al. Early risk stratification in patients with cardiogenic shock irrespective of the underlying cause - the Cardiogenic Shock Score. Eur J Heart Fail. 2022;24(4):657-67.

8. Harjola VP, Lassus J, Sionis A, Kober L, Tarvasmaki T, Spinar J, et al. Clinical picture and risk prediction of short-term mortality in cardiogenic shock. Eur J Heart Fail. 2015;17(5):501-9.

9. Arias FG, Alonso-Fernandez-Gatta M, Dominguez MP, Martinez JM, Veloso PR, Bermejo RMA, et al. Predictive Model and Risk Score for In-Hospital Mortality in Patients with All-Cause Cardiogenic Shock. Int Heart J. 2022.

10. Barthelemy O, Rouanet S, Brugier D, Vignolles N, Bertin B, Zeitouni M, et al. Predictive Value of the Residual SYNTAX Score in Patients With Cardiogenic Shock. J Am Coll Cardiol. 2021;77(2):144-55.

11. Champion S. Toward catecholamine responsiveness in cardiogenic shock: insights from the CRASH score. Int J Artif Organs. 2016;39(2):94-7.
